# Endovascular Versus Open Surgical Approaches for Acute Mesenteric Ischemia: A Systematic Review of Outcomes

**DOI:** 10.7759/cureus.85013

**Published:** 2025-05-29

**Authors:** Mohammad Nazir Zaman, Wahida Ali, Wahidullah Dost, Mohammad Qaher Rasully, Raisa Dost, Jamaluddin Niazi, Wahida Dost, Farzad Qasemi

**Affiliations:** 1 General Surgery, Jamhuriat Hospital, Kabul, AFG; 2 Surgery and Medicine, Kabul University of Medical Sciences, Kabul, AFG; 3 Cardiac Surgery, Kabul University of Medical Sciences, Kabul, AFG; 4 Cardiovascular Surgery, Punjab Institute of Cardiology, Lahore, PAK

**Keywords:** acute mesenteric ischemia, endovascular therapy, superior mesenteric artery, thromboembolic occlusion, vascular emergency

## Abstract

Acute mesenteric ischemia (AMI) remains a life-threatening vascular emergency, with significant clinical challenges despite advances in diagnostic and therapeutic approaches. This systematic review compared the efficacy and outcomes of endovascular versus open surgical interventions in AMI management. A comprehensive search of PubMed/MEDLINE, Embase, Web of Science, and the Cochrane Library was conducted from database inception through February 15, 2025, yielding six retrospective cohort studies that met inclusion criteria. Endovascular intervention was associated with lower in-hospital mortality compared to open surgery in most studies. Patients undergoing endovascular therapy demonstrated significantly lower bowel resection rates and preserved intestinal length. Additional benefits included shorter hospital stays, reduced ICU length of stay, and fewer complications, including acute renal failure and pulmonary complications. However, endovascular approaches were associated with higher reintervention rates. Independent predictors of mortality included elevated lactate concentration, pneumatosis intestinalis, increased white blood cell count, chronic renal insufficiency, and extensive bowel necrosis. Endovascular therapy represents a promising approach for AMI management, particularly in patients without evidence of bowel necrosis at presentation, though careful patient selection and vigilant monitoring are essential due to higher reintervention rates. Prospective studies are needed to better define optimal treatment strategies for different AMI patient subgroups.

## Introduction and background

Acute mesenteric ischemia (AMI) is a life-threatening condition characterized by the sudden interruption of blood supply to a segment of the intestine, leading to ischemia, cellular damage, and potential intestinal necrosis if untreated. It accounts for 0.1% to 0.2% of hospitalizations and is a rare, yet deadly, vascular emergency [1]. AMI can be classified as occlusive, caused by mesenteric arterial embolism (50%), arterial thrombosis (15%-25%), or venous thrombosis (5%-15%), or as non-occlusive mesenteric ischemia (NOMI) [2,3]. Diagnosis is challenging due to nonspecific symptoms and vague abdominal pain, often delaying treatment and increasing mortality risk [1,2]. Superior mesenteric artery embolism (SMAE) is the leading cause of AMI, typically arising from cardiac rhythm disorders such as atrial fibrillation, thrombus formation in the left atrium, aortic plaque buildup, pre-existing vascular narrowing, or malignancies [4].

The management of AMI depends on the nature, acuity, and severity of the disease. Early intervention focuses on resecting the nonviable bowel, restoring blood flow to the ischemic intestine, and providing supportive care. In acute thromboembolic events, operative embolectomy has been the traditional approach, offering favorable short- and long-term outcomes. Surgical revascularization for visceral ischemia due to an occluded superior mesenteric artery (SMA) was first described by Shaw and Maynard in 1958, with successful cases of mesenteric thromboendarterectomy [5,6].

With ongoing advancements in surgery, endovascular therapy has emerged as a significant innovation, gaining prominence since the first reports of percutaneous transluminal angioplasty of the visceral vessels in 1980 [6]. In both acute and chronic intestinal ischemia, endoluminal therapy has emerged as a primary treatment option. Stent placement has shown symptom relief, particularly in the proximal celiac artery. Along with the advancement of endovascular therapy, incorporating adjunct therapies, such as antiplatelet treatment, has significantly reduced the thrombosis rate of mesenteric prostheses from 18% to 1% [7].

Both open surgery and endovascular procedures offer unique advantages and challenges in managing mesenteric ischemia. Open surgery typically provides more durable long-term results, though it carries a higher risk of periprocedural mortality. On the other hand, endovascular therapy reduces the need for laparotomies and serves as a valuable option for high-risk patients, helping to lower mortality rates. However, it also has drawbacks, including a higher likelihood of recurrent symptoms and restenosis, often requiring additional interventions [6].

As experience with high-volume endovascular centers continues to grow, so too will our understanding of the optimal use of these techniques. Emerging prospective data is expected to provide clearer insights into the efficacy of endovascular therapy, particularly in comparison to open surgery, and will help refine treatment strategies for different patient populations. For now, both open surgery and endovascular interventions offer complementary options that, when carefully considered, can provide the best outcomes for patients with AMI, depending on their individual clinical presentation and overall risk profile.

## Review

Materials and methods

Search Strategy

This systematic review was conducted in accordance with the Preferred Reporting Items for Systematic Reviews and Meta-Analyses (PRISMA) 2020 guidelines [8]. We performed a comprehensive literature search across multiple electronic databases, including PubMed/MEDLINE, Embase, Web of Science, and the Cochrane Library. The search period encompassed all articles published from database inception through February 15, 2025. The search strategy combined terms related to AMI ("acute mesenteric ischemia," "intestinal ischemia," "bowel ischemia") with terms related to surgical interventions ("open surgery," "laparotomy," "bowel resection," "endovascular treatment," "angioplasty," "stenting," "thrombolysis"). Additional relevant articles were identified through manual searching of reference lists from included studies and relevant review articles.

Eligibility Criteria

Studies were eligible for inclusion if they met the following criteria: original research articles published in peer-reviewed journals; study designs including randomized controlled trials (RCTs), cohort studies, and case-control studies; studies comparing open surgical and endovascular approaches for AMI; studies reporting quantitative measures of clinical outcomes, such as mortality, bowel viability, reintervention rates, length of hospital stay, and procedural complications; studies conducted in human subjects; and articles published in English. Exclusion criteria comprised review articles, editorials, letters, and conference abstracts; studies focusing solely on chronic mesenteric ischemia; animal or in vitro studies; studies without clear documentation of comparative surgical outcomes; and studies lacking quantitative assessment of patient outcomes.

Data Extraction

Two independent reviewers (W.A. and W.D.) extracted data using a standardized form developed for this review. The following information was collected from each study: author, publication year, country, study design, sample size, patient demographics, AMI etiology (embolic, thrombotic, or non-occlusive), intervention details (open surgery or endovascular procedure type), primary outcomes (mortality, bowel viability, reintervention rates), and secondary outcomes (length of hospital stay, procedural complications, long-term survival). Discrepancies in data extraction were resolved through discussion with a third reviewer (J.N.).

Statistical Analysis

Due to potential heterogeneity in study designs, intervention techniques, and outcome assessments, we conducted a qualitative synthesis of the evidence rather than a meta-analysis. We evaluated the consistency of findings across studies, considering the strength of associations, differences in surgical technique, and potential confounders. Where possible, we assessed the impact of study quality, follow-up duration, and adjustment for confounding factors on the observed outcomes.

Results

Study Selection Process

**Figure 1:**
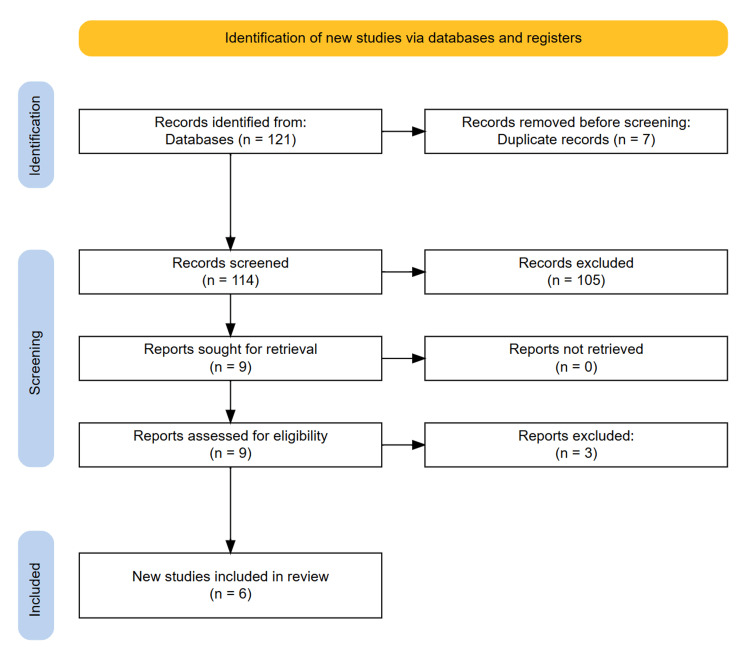
PRISMA diagram showing the study selection process PRISMA, Preferred Reporting Items for Systematic Reviews and Meta-Analyses

The study selection process was conducted in accordance with PRISMA guidelines to ensure a transparent and systematic approach. An initial comprehensive search identified 121 studies. After the removal of seven duplicate records, 114 unique studies remained. Titles and abstracts were screened, leading to the exclusion of 105 studies that did not meet the predefined relevance criteria. Full-text evaluation of the remaining nine articles resulted in the exclusion of three studies that did not meet the stringent inclusion criteria. Ultimately, six studies were deemed eligible for inclusion in the systematic review. The detailed study selection process is illustrated in the PRISMA flowchart (Figure 1).

Study Characteristics

A total of six retrospective cohort studies, including 951 patients, were analyzed (Table 1). The sample sizes ranged from 25 to 679, with a mean patient age between 45 and 73 years. The majority of patients presented with acute thromboembolic occlusion of the SMA, while some studies also included cases of acute superior mesenteric venous thrombosis and NOMI. The most common comorbidities across studies were hypertension, atrial fibrillation, diabetes, and coronary artery disease.

**Table 1:** Summary of the main findings of included studies AMI, Acute Mesenteric Ischemia; ASMVT, Acute Superior Mesenteric Venous Thrombosis; ATOS, Acute Thromboembolic Occlusion of the Superior Mesenteric Artery; ARDS, Acute Respiratory Distress Syndrome; ASA, American Society of Anesthesiologists; CAD, Coronary Artery Disease; CI, Confidence Interval; ICU, Intensive Care Unit; IQR, Interquartile Range; MI, Myocardial Infarction; NOMI, Non-occlusive Mesenteric Ischemia; OR, Odds Ratio; PAD, Peripheral Arterial Disease; RMB, Renminbi, Chinese currency; SMAE, Superior Mesenteric Artery Embolism; SMA, Superior Mesenteric Artery; TPN, Total Parenteral Nutrition; WBC, White Blood Cell

Author	Year of publication	Country	Study design	Sample size (open surgery/endovascular)	Patient demographics	AMI etiology	Outcomes studied	Main findings of primary outcomes	Main findings of secondary outcomes	Summary
Beaulieu et al. [1]	2014	United States	Retrospective cohort	679 (514/165)	Mean age: 70.5 years; 57.1% female; No significant difference in comorbidities between treatment groups (as measured by Charlson index); Open group had higher rates of lactic acidosis (30.0% vs. 11.4%) and ARDS (31.5% vs. 7.0%)	Included all AMI etiologies (embolic, thrombotic, and nonocclusive disease), though specific breakdown not provided	Primary: Mortality, Length of stay. Secondary: Need for bowel resection, Requirement for total TPN	Mortality was significantly lower with endovascular treatment (24.9% vs 39.3%, p=0.01) - Length of stay was significantly shorter with endovascular treatment (12.9 vs. 17.1 days, p = 0.006) - Time from admission to procedure was similar between groups (1.86 days for endovascular vs. 1.99 days for open, p = 0.72)	Bowel resection rates were significantly lower with endovascular treatment (14.4% vs. 33.4%, p < 0.001) - TPN requirement was significantly lower with endovascular treatment (13.7% vs. 24.4%, p = 0.025)	This study demonstrated that endovascular intervention for AMI has increased significantly from 2005 to 2009 (11.9% to 30.0%). Among AMI patients undergoing revascularization, endovascular treatment was associated with decreased mortality, shorter length of stay, lower rates of bowel resection, and reduced need for TPN compared to open surgery. These findings suggest endovascular approaches may improve outcomes in AMI patients, though patient selection factors may have influenced the results.
Li et al. [4]	2022	China	Retrospective cohort	41 (27/14)	Median age: 70 years (IQR 60-77); 56.1% male; 71% with atrial fibrillation; 44% with diabetes; 66% with hypertension; 44% with coronary artery disease	SMAE	Technical success, surgery duration, blood loss, bowel resection rate, length of resected bowel, complications, mortality	Technical success of endovascular treatment: 93% - Endovascular treatment had shorter surgery duration (102 vs. 210 min, p < 0.05) - Endovascular treatment had less blood loss (20 vs. 200 mL, p < 0.05) - No significant difference in mortality between groups (21% vs. 33%, p > 0.05)	Endovascular group had shorter bowel rest time (6 vs. 11 days, p = 0.022) - Endovascular group had shorter ICU stay (1 vs. 5 days, p = 0.004) - Endovascular group required less ventilator use (0 vs. 11 hrs, p = 0.011) - Endovascular group had higher reoperation rate (36% vs. 4%, p < 0.02) - Predictors of mortality: chronic renal insufficiency, ASA > 3, WBC > 12 × 10³/dL, creatinine > 92 μmol/dL, urea > 6.2 mmol/L, necrotic bowel > 2 m	Endovascular treatment is feasible for SMAE patients without clinical evidence of bowel necrosis. Compared to open surgery, it offers advantages in surgery duration, blood loss, bowel rest time, ICU stay, and ventilator use, but with higher reoperation rates. Mortality rates were similar between the two approaches. Patient selection is crucial, as those with initial elevated WBC and neutrophil counts were at higher risk for bowel necrosis and may benefit from open surgery.
Li et al. [9]	2024	China	Retrospective cohort	62 (37/21 plus 4 conservative treatment)	Median age: 69 years (IQR 58-79); 55% male; Comorbidities: hypertension (69%), atrial fibrillation (66%), diabetes (34%), coronary artery disease (35%)	ATOS: 73% embolic, 27% thrombotic	Technical success rate, 30-day mortality, procedure duration, blood loss, ischemic bowel requiring resection, complications (acute kidney injury, pulmonary failure, MI, stroke), second-look operations, sepsis, hospital/ICU stay, ventilator time	Technical success: endovascular (90.5%) vs. open surgery (97.3%). No significant difference in 30-day mortality between groups (19% endovascular vs. 43% open surgery, p = 0.200) - Endovascular had shorter procedure time (95 vs. 200 min, p < 0.01) and less blood loss (20 vs. 200 mL, p < 0.01)	Less bowel resection needed in endovascular group (35% vs. 70%, p = 0.020) - Endovascular group had shorter bowel rest time, ICU stay, and ventilator use - Higher second-look operation rate in endovascular group (33% vs. 3%, p = 0.004) - Trend of higher sepsis in open surgery group	Endovascular or conservative treatment may be suitable for selected patients without signs of bowel necrosis, with close monitoring for bowel necrosis. Independent risk factors for 30-day mortality were: neutrophil count >12 × 10³/dL, age >60 years, and history of chronic renal insufficiency.
Kapalla et al. [10]	2023	Germany	Retrospective cohort	74 (61/13)	Mean age: 73.6 ± 11.7 years, 72% male; Common comorbidities: hypertension (88%), atrial fibrillation (49%), diabetes mellitus (43%), peripheral arterial occlusive disease (34%)	49% arteriosclerotic, 51% thromboembolic	Primary: In-hospital mortality, Secondary: Technical success, morbidity, long-term survival, revision rates	In-hospital mortality: 43.3% overall - Open treatment: 41% - Endovascular: 53.8% (p = 0.54, no significant difference)	High complication rate (85.1%) - Common complications: sepsis (56.8%), acute kidney injury (44.6%), pulmonary complications (28.4%) - 29.7% required additional bowel resection - Mean follow-up: 19 months to 1-year survival: 85.5% - 5-year survival: 58.8%	Morbidity and mortality of AMI remain high. Conventional open or intraoperative endovascular therapy achieved similar results in patients requiring laparotomy. Advanced disease stage with ischemic intestinal sections at presentation and cardiovascular comorbidities were associated with adverse outcomes. Independent risk factors for mortality were pneumatosis intestinalis, increased lactate concentration, and ischemic intestinal sections.
Yang et al. [11]	2014	China	Retrospective cohort	25 (12/13)	Mean age: 45.08 ± 11.74 years; 15 males, 10 females	ASMVT	Primary: length of bowel resection, time to symptom elimination, time to enteral/oral nutrition, hospital stay, total cost. Secondary: morbidity, 30-day mortality, 1-year survival	The endovascular group had significantly shorter time to symptom elimination (7.23 ± 2.42 days vs. 18.25 ± 7.69 days), earlier enteral/oral nutrition (8.92 ± 1.89 days vs. 20.50 ± 5.13 days), and shorter hospital stay (20.46 ± 6.59 days vs. 43.00 ± 13.77 days). The length of bowel resection was significantly less in the endovascular group (median 0 cm vs 155 cm). Total cost was lower in the endovascular group (72,785.6 ± 21,828.16 RMB vs. 200,020.4 ± 91,505.62 RMB)	No significant difference in 30-day mortality (both groups had high survival) and 1-year survival (92.3% vs. 75%). The endovascular group had no cases of short bowel syndrome compared to 5 cases (41.7%) in the surgical group. No significant differences in complications such as bleeding, acute kidney injury, or secondary operations	For patients with ASMVT and circumscribed peritonitis, initial transcatheter thrombolysis demonstrated better outcomes than prompt surgical exploration in terms of conserving bowel length, faster symptom resolution, earlier nutrition restoration, shorter hospital stay, and lower cost, while maintaining similar survival rates. Early diagnosis and prompt intervention were key factors for successful outcomes.
Arthurs et al. [12]	2011	USA	Retrospective cohort	70 (14/56)	Mean age: 64 ± 13 years; 50% male; Comorbidities: hypertension (80%), diabetes (32%), hyperlipidemia (44%), smoking (28%), chronic renal failure (12%), PAD (35%), CAD (41%), atrial fibrillation (26%)	65% thrombotic (80% native vessel thrombosis, 16% stent occlusions, 4% iatrogenic); 35% embolic (85% cardiac source, 15% from cardiac procedures)	In-hospital mortality, endovascular technical success, operative complications, length of bowel resection, need for laparotomy	Successful endovascular treatment resulted in a mortality rate of 36% vs. 50% with traditional therapy (p < 0.05); Endovascular therapy was associated with improved mortality in thrombotic AMI (OR 0.10; 95% CI, 0.10-0.76; p < 0.05); Overall technical success for endovascular therapy was 87%	Endovascular therapy required laparotomy in 69% vs. traditional therapy in 100% (p < 0.05); Median 52 cm necrotic bowel resected with endovascular vs. 160 cm with traditional therapy (p < 0.05); Acute renal failure and pulmonary failure occurred less frequently with endovascular therapy (27% vs. 50% and 27% vs. 64%, respectively; p < 0.05)	Endovascular therapy has altered the management of AMI with measurable advantages. Using endovascular therapy as the primary modality for AMI reduces complications and improves outcomes, particularly for thrombotic occlusions. The study represents the largest single-center experience using endovascular therapy for AMI treatment at the time of publication.

Endovascular intervention was associated with a lower in-hospital mortality rate compared to open surgery in most studies. Beaulieu et al. reported significantly lower mortality with endovascular treatment, while Li et al. found a 30-day mortality of 19% in endovascular vs. 43% in open surgery (p = 0.2) [1,9]. However, Kapalla et al. observed no significant difference, highlighting that patient selection and disease severity may influence outcomes [10]. Some studies indicated a lower bowel resection rate in the endovascular group. Yang et al. reported a median bowel resection of 0 cm vs. 155 cm in favor of endovascular treatment [11]. Similarly, Beaulieu et al. found a significantly lower resection rate in the endovascular group, while Li et al. showed that 35% of endovascular patients required resection, compared to 70% in open surgery (p = 0.02) [1,9].

Patients undergoing endovascular therapy had a significantly shorter hospital stay. Beaulieu et al. reported 12.9 vs. 17.1 days (p = 0.006), and Yang et al. found a 20.46-day stay for endovascular vs. 43.00 days for open surgery [1,11]. Endovascular therapy was associated with fewer complications, such as acute renal failure and pulmonary complications. Arthurs et al. found lower rates of acute renal failure and pulmonary failure in the endovascular group [12]. However, Kapalla et al. reported high morbidity rates for both groups, with complications including sepsis and acute kidney injury [10]. Reintervention rates were higher for the endovascular group. Li et al. found that 33% of endovascular patients required a second-look operation, compared to only 3% in open surgery (p = 0.004) [9]. Kapalla et al. also reported a higher revision rate with endovascular therapy [10].

Patients in the endovascular group had shorter ICU stays and reduced ventilator dependency. Li et al. reported a median ICU stay of one day in the endovascular group vs. five days in open surgery (p = 0.004), with significantly lower ventilator use [4]. Independent predictors of mortality included elevated lactate concentration, presence of pneumatosis intestinalis, increased WBC count (>12 × 10³/dL), chronic renal insufficiency, and extensive bowel necrosis (>2 meters) [4].

Discussion

AMI remains one of the most challenging vascular emergencies, with high mortality rates when diagnosis and treatment are delayed. The pathophysiology of AMI involves sudden interruption of blood supply to intestinal segments, leading to cellular hypoxia, mucosal barrier disruption, bacterial translocation, and, ultimately, intestinal necrosis if not promptly addressed [13]. SMAE represents the most common etiology, typically originating from cardiac sources such as atrial fibrillation or left atrial thrombi, while arterial thrombosis generally occurs in patients with pre-existing atherosclerotic disease [14].

The diagnostic challenge in AMI stems from its nonspecific early presentation, which often includes vague abdominal pain disproportionate to physical examination findings. This diagnostic uncertainty frequently leads to treatment delays that significantly impact survival outcomes. Modern diagnostic approaches rely heavily on contrast-enhanced CT angiography, which has become the gold standard for non-invasive diagnosis, with high sensitivity and specificity [15]. Laboratory markers, such as elevated lactate, D-dimer, and inflammatory parameters, can support the diagnosis but lack specificity in the early stages. This systematic review of six retrospective cohort studies encompassing 951 patients provides important insights into the evolving management strategies for AMI. The traditional approach - involving open surgical exploration, thromboembolectomy, and bowel resection when necessary - has been progressively complemented by endovascular techniques since the first reports of percutaneous transluminal angioplasty of visceral vessels in 1980. Our analysis demonstrates that endovascular interventions offer several potential advantages over conventional open surgery in selected patients.

Most notably, endovascular therapy was associated with lower in-hospital mortality compared to open surgery in four of the six studies, with Beaulieu et al. reporting a significant reduction from 39.3% to 24.9% (p = 0.01) [1]. This mortality benefit was particularly pronounced in patients with thrombotic occlusions, as demonstrated by Arthurs et al. (OR 0.10; 95% CI, 0.10-0.76; p < 0.05) [12]. The endovascular approach also appears to preserve more viable bowel tissue, with significantly lower bowel resection rates across multiple studies. Yang et al. reported a striking difference in median bowel resection length (0 cm vs. 155 cm), while Li et al. found a significantly lower proportion of patients requiring resection in the endovascular group (35% vs. 70%, p = 0.020) [9,11].

The perioperative benefits of endovascular intervention were consistent across studies, with significantly shorter procedure times, reduced blood loss, decreased hospital and ICU length of stay, and less ventilator dependency. These advantages likely reflect the less invasive nature of endovascular approaches and may be particularly beneficial for high-risk patients with significant comorbidities who might not tolerate extensive open surgery.

However, several important limitations of endovascular therapy were identified. The higher reintervention rates observed in the endovascular groups (33% vs. 3%, p = 0.004 in Li et al.) highlight the potential need for vigilant postprocedural monitoring and readiness for secondary interventions [9]. This finding suggests that, while endovascular approaches may provide initial technical success, they may not always provide definitive treatment. The requirement for second-look operations underscores the need for careful patient selection and close monitoring for signs of ongoing or recurrent intestinal ischemia following endovascular procedures.

Importantly, patient selection emerges as a critical factor in determining appropriate management strategies. Li et al. identified elevated lactate levels, pneumatosis intestinalis, neutrophil count >12 × 10³/dL, chronic renal insufficiency, and extensive bowel necrosis as independent predictors of mortality [4]. These factors should inform clinical decision-making when choosing between open surgical and endovascular approaches. Patients presenting with clinical or radiological evidence of bowel necrosis may benefit more from immediate open surgical intervention to facilitate bowel assessment and resection, while those with earlier presentation and without evidence of necrosis may be suitable candidates for endovascular approaches. The integration of both treatment modalities through hybrid approaches represents a promising direction for AMI management. As demonstrated by Kapalla et al., intraoperative endovascular therapy during open surgical exploration can combine the benefits of direct bowel assessment with less invasive revascularization techniques [10]. This hybrid approach may be particularly valuable in cases where the viability of intestinal segments remains uncertain, despite successful revascularization.

Despite the apparent advantages of endovascular therapy in selected patients, several limitations of the included studies must be acknowledged. The retrospective nature of all six studies introduces potential selection bias, with endovascular treatment likely preferentially offered to patients with less severe disease or earlier presentation. Additionally, the heterogeneity in patient characteristics, AMI etiologies, and specific endovascular techniques limits the generalizability of these findings. Well-designed prospective studies, with standardized protocols, are needed to definitively establish the optimal role of endovascular therapy in AMI management.

## Conclusions

This systematic review highlights the evolving role of endovascular therapy in AMI, offering benefits such as lower in-hospital mortality, reduced bowel resection rates, shorter hospital stays, and fewer complications compared to open surgery. These advantages are most pronounced in patients with thromboembolic occlusions without advanced intestinal necrosis. However, higher reintervention rates necessitate careful patient selection and close monitoring. Identifying key mortality predictors - elevated lactate levels, pneumatosis intestinalis, leukocytosis, and chronic renal insufficiency - can aid clinical decision-making. Hybrid approaches combining open and endovascular techniques may optimize outcomes, especially in cases of uncertain bowel viability. Despite these benefits, study limitations such as retrospective designs and selection bias prevent definitive conclusions. Prospective trials are essential to establish evidence-based guidelines. Until then, a multidisciplinary approach involving vascular surgeons, interventional radiologists, and critical care teams remains crucial for managing this complex vascular emergency.
